# Multiple insecticide resistance in the major malaria vector *Anopheles funestus* in southern Ghana: implications for malaria control

**DOI:** 10.1186/s13071-016-1787-8

**Published:** 2016-09-15

**Authors:** Jacob M. Riveron, Michael Osae, Alexander Egyir-Yawson, Helen Irving, Sulaiman S. Ibrahim, Charles S. Wondji

**Affiliations:** 1 Vector Biology Department, Liverpool School of Tropical Medicine, Pembroke Place, Liverpool, UK; 2 Research Unit Liverpool School of Tropical Medicine, Organisation de Coordination pour la lutte contre les Endémies en Afrique Centrale, P.O Box 288, Yaoundé, Cameroon; 3 Ghana Atomic Energy Commission, Accra, Ghana; 4 Department of Biochemistry, Bayero University, PMB 3011 Kano, Nigeria

**Keywords:** Malaria, Insecticide resistance, Vector control, *An. funestus*, *An. gambiae*, *An. coluzzii*, Ghana

## Abstract

**Background:**

Understanding the dynamics of insecticide resistance in African malaria vectors is crucial for successful implementation of resistance management strategies in the continent. This study reports a high and multiple insecticide resistance in *Anopheles funestus* from southern Ghana which could compromise the Malaria Operational Plan in this country, if not tackled. Adult *Anopheles* mosquitoes were collected in Obuasi and Adawukwa, in southern Ghana. *Plasmodium* infection rates, susceptibility to the main insecticides used in public health and the molecular basis of insecticide resistance were established.

**Results:**

*An. funestus* (*sensu stricto*) (*s.s*.) was the predominant mosquito species found resting inside the houses in Obuasi, while at Adawukwa it was found together with *An. coluzzii*. Parasite rates were high in *An. funestus* (*s.s*.) populations from both localities, with *Plasmodium* infection rates greater than 12.5 %. Both, *An. funestus* (*s.s*.) and *An. coluzzii*, from the two sites exhibited high resistance to the insecticide from various classes including the pyrethroids, carbamates and DDT, but remained fully susceptible to the organophosphates. A preliminary characterization of the underlying molecular mechanisms of resistance in *An. funestus* (*s.s*.) populations from both sites revealed that *CYP6P9a*, *CYP6P9b*, *CYP6M7* and *GSTe2* genes are upregulated, markedly higher in Obuasi (between 3.35 and 1.83 times) than in Adawukwa population. The frequency of L119F-GSTe2 and A296S-RDL resistance markers were also higher in Obuasi (42.5 and 68.95 % higher), compared with *An. funestus* (*s.s*.) populations from Adawukwa. These findings suggest that the similar resistance pattern observed in both *An. funestus* (*s.s*.) populations are driven by different mechanisms.

**Conclusions:**

Resistance to multiple insecticides in public health use is present in malaria vectors from Ghana with major resistance genes already operating in the field. This should be taken into consideration in the design of resistance management strategies to avoid operational failure.

## Background

Malaria is endemic in Ghana with the entire population of 24.2 million at risk of infection and more than 3 million cases of clinical malaria reported annually, of which 900,000 cases are in children under the age of five [1]. To reduce this burden, the President’s Malaria Initiative (PMI), in collaboration with Ghana National Malaria Control Program and other partners, has developed the PMI/Ghana Malaria Operational Plan (MOP). Besides early diagnosis, intermittent preventive treatment of pregnant women and artemisinin-based combination therapies, MOP targets malaria vectors through free distribution of long-lasting insecticide-treated nets (LLINs) and a progressive scale up of indoor residual spraying (IRS) campaigns. Unfortunately, increasing insecticide resistance in malaria mosquitoes to the main insecticides used for both LLINs and IRS in Ghana and other African countries [2, 3] is threatening the continued effectiveness of these interventions.

Insecticide resistance in malaria vectors is a dynamic process where the resistance pattern might change quickly because of selection pressures from both public health and agricultural practices [4, 5]. Implementation of successful resistance management strategies requires up-to-date information of insecticide resistant patterns in malaria vectors, as advised by the WHO Global Plan for Insecticide Resistance Management in Malaria Vectors [6], in order to utilise appropriate insecticides, and also to establish the molecular mechanisms driving the resistance.

In Ghana, *An. funestus* (*sensu stricto*) (*s.s*.) along with *An. gambiae* (*sensu lato*) (*s.l*.), are the most important vectors of malaria, with *An. funestus* (*s.s*.) being prevalent in some areas of the country [7]. Several studies carried out between 2004 and 2010 throughout Ghana have shown that *An. funestus* (*s.s*.) populations are fully susceptible to deltamethrin (a type II pyrethroid), commonly used in LLINs and IRS, and to the organophosphate malathion. However, resistance to permethrin (a type I pyrethroid), also commonly used in LLINs and IRS, was detected in *An. funestus* (*s.s*.) for the first time in Obuasi, southern Ghana, in 2005 [8]. Resistance to other classes of insecticides used in public health such as the organochlorine dichlorodiphenyltrichloroethane (DDT) and the carbamate bendiocarb, was also reported in the same location in 2004 [2]. With no knockdown resistance (*kdr*) mutation detected so far in *An. funestus*, previous studies have demonstrated that pyrethroids and DDT resistance results from an increase in insecticide metabolism catalyzed mainly by the cytochrome P450s and glutathione S-transferases, with *CYP6P9a*, *CYP6P9b*, *CYP6M7* and *GSTe2* playing the key roles [9–11]. Beside the overexpression of these enzymes, it is acknowledged that the presence of L119F-GSTe2 mutation confers DDT resistance in *An. funestus* (*s.s*.) populations in West/Central and East Africa, as the 119 F-GSTe2 enzyme is 3.4 times more efficient at metabolizing DDT in vitro than the L119-GSTe2 wild-type form [11]. However, the molecular mechanisms of insecticide resistance in Ghana remain uncharacterized.

To assist the efforts of malaria vector control and help in developing effective resistance management plans, this study reports the contribution to malaria transmission and the insecticide resistant profile of two *An. funestus* (*s.s*.) populations collected in two districts of southern Ghana, Obuasi and Adawukwa, in 2014. Furthermore, the molecular mechanisms of the resistance are investigated by assessing the expression profile of the major metabolic resistance genes in this species (*CYP6P9a*, *CYP6P9b*, *CYP6M7* and *GSTe2*) and the presence of L119F-GSTe2- and A296S-RDL-resistant mutations. Beside these two populations, the insecticide resistant profiles and the *Plasmodium* infection rate of one *An. coluzzii* population from Adawukwa, collected at the same time, were also characterized.

## Methods

### Study area and mosquito collection

Adult female A*nopheles* mosquitoes were collected in two localities in southern Ghana, Atatam village (5°56′N, 1°37′W) in the Adansi North District, close to Obuasi Municipality of the Ashanti Region of Ghana, and Adawukwa (5°24′N, 0°36′W) in the Gomoa East District of the Central Region of Ghana, close to Accra, the capital city, in March and November 2014, respectively. These areas have tropical climate, with two wet seasons, one in March to July and a shorter wet season in September to November. The mean annual temperature is 26 °C and the mean annual rainfall is approximately 790 mm. Detailed information on these study sites are presented in [12].

After obtaining consent from village chiefs and house owners, indoor resting mosquitoes were collected inside households in the morning, usually until midday, using battery-operated insect aspirators. Blood-fed and female mosquitoes collected were kept in cages until they become fully gravid. The mosquitoes were morphologically identified as belonging to either the *An. funestus* group or *An. gambiae* complex according to a morphological key [13] and forced to lay eggs in individual 1.5 ml micro-centrifuge tubes as described previously [14]. Due to the low number of *An. gambiae* (*s.l*.) collected in Obuasi, only the population from Adawukwa was characterized. Egg batches and dead F_0_ adult female mosquitoes were transported to the Liverpool School of Tropical Medicine (Liverpool, UK) under the DEFRA license (PATH/125/2012, Department for Environment, Food and Rural Affairs, UK).

### Species identification

gDNA was extracted from whole F_0_ female mosquitoes using the DNeasy Blood and Tissue kit (Qiagen, Hilden, Germany), and utilized in a cocktail PCR to identify the different species within the *An. funestus* group and *An. gambiae* complex as previously described [15, 16]. Subsequently, the gDNA extracted was utilized to determinate the *Plasmodium* infection rates and to genotype the resistance markers. F_1_ egg batches from individual parent females were hatched in small paper cups and larvae were then transferred to plastic trays for rearing, as previously described [14, 17].

### *Plasmodium* infection rates

Using 40 F_0_ female mosquitoes from each location, the *Plasmodium* infection rates in both Obuasi and Adawukwa *An. funestus* populations were determined by a TaqMan assay as previously described [18, 19]. This method detects the presence of *Plasmodium falciparum* and/or *P. ovale*, *P. vivax* and *P. malariae* (OVM+). The *Plasmodium* infection rate of the *An. gambiae* (*s.l*.) population from Adawukwa was also determined using 46 F_0_ female mosquitoes. Afterwards, all positive samples were validated by a nested PCR [20].

### WHO insecticide susceptibility assays

WHO bioassays, using insecticide-impregnated papers, were performed to determine the susceptibility profile of the different populations collected against four classes of insecticides used in public health: the pyrethroids type I permethrin (0.75 %) and type II deltamethrin (0.05 %, only for Adawukwa populations), the carbamate bendiocarb (0.1 %), and the organochlorine DDT (4 %). Due the low number of samples from Obuasi, the organophosphates malathion (5 %) was only used for mosquitoes from Adawukwa. F_1_ male and female adults, 2–5 day-old were exposed to the insecticide-treated papers for 60 min and then transferred to holding tubes with access to 10 % sugar solution, as described in the WHO manual [21]. Mortality rate was determined 24 h after exposure. Assays were carried out with at least 4 replicates of 20–25 mosquitoes each, at 25 ± 1 °C and 70–80 % relative humidity. For each bioassay, control mosquitoes were also exposed to papers impregnated with only insecticide carrier oil following the same procedure.

Furthermore, the insecticide resistant profile of one *An. gambiae* population from Adawukwa, sympatric with the *An. funestus* population, was also characterized by WHO insecticide susceptibility assays using permethrin (0.75 %), deltamethrin (0.05 %), bendiocarb (0.1 %), DDT (4 %), malathion (5 %) and pirimiphos-methyl (1 %). Due to the high resistance shown by Adawukwa mosquitoes against permethrin and in order to estimate the intensity of this resistance, additional bioassays were performed where F_1_ female mosquitoes were exposed to 0.75 % permethrin-treated papers for 120, 240 and 360 min to determine LT_50_.

Synergist assay with piperonyl butoxide (PBO) was also performed for *An. gambiae.* F_1_ adults from Adawukwa with pre-exposure to 4 % PBO impregnated paper for 1 h followed immediately with exposure to permethrin (0.75 %) or DDT (4 %) for another 1 h. Mortality rate was scored after 24 h. Due to the low number of adult F_1_*An. funestus* (*s.s*.) mosquitoes, synergist assays were not performed for this species.

### Transcription profile of the major resistance genes

The expression profiles of *CYP6P9a*, *CYP6P9b* and *CYP6M7*, previously associated with pyrethroid resistance in *An. funestus* (*s.s*.) populations [9, 10], and *GSTe2*, associated with DDT resistance in *An. funestus* (*s.s*.) in West Africa [11] was assessed by qRT-PCR as previously described [5]. Total RNA was extracted from three batches of ten F_1_ female mosquitoes for three conditions: non-exposed to insecticides, permethrin- and DDT-resistant, in both localities; and for the fully susceptible laboratory strain FANG. RNA extraction, cDNA synthesis and qRT-PCR reactions and analysis were conducted as previously described [5, 22]. The relative expression and fold-change of the four target genes were calculated according to 2^-ΔΔCT^ method [23], after normalization with housekeeping genes *Actin* (VectorBase ID: AFUN006819-RA) and *RSP7* (ribosomal protein S7; VectorBase ID: AFUN007153-RA). Differences in expression were statistically analysed using unpaired Student’s *t*-test.

### Genotyping of L119F-GSTe2 and A296S-RDL resistance markers

To assess the role of the L119F-GSTe2 mutation in DDT resistance in Obuasi and Adawukwa, a TaqMan assay was used to genotype 40 F_0_ field-collected females, as described previously [11]. Furthermore, the A296S-RDL mutation conferring dieldrin resistance was also genotyped by a TaqMan assays [5] using the same female mosquitoes.

## Results

### Species identification

Results from PCR-species identification performed from F_0_ females morphologically identified as *An. funestus* group in Obuasi and Adawukwa, revealed that they all belong to the major malaria vector *An. funestus* (*s.s*.). For mosquitoes morphologically identified as *An. gambiae* complex in Adawukwa, the F_0_ females were all *An. coluzzii*. Only mosquitoes belonging to the *An. funestus* group were collected in Obuasi.

### *Plasmodium* infection rate

Forty F_0 _*An. funestus* (*s.s*.) females from both localities, and 46 F_0 _*An. coluzzii* females from Adawukwa were screened for *P. falciparum* and *P. ovale/P. vivax/P. malariae* (OVM+) using TaqMan assay. The *Plasmodium* infection rates for *An. funestus* (*s.s*.) populations from Obuasi and Adawukwa were similar (12.5 %, 5/40). Four mosquitoes infected with *P. falciparum* (10 %, 4/40) and one with OVM+ (2.25 %, 1/40) were detected in Obuasi while only *P. falciparum* (12.5 %, 5/40) were detected in mosquitoes from Adawukwa. A nested PCR performed with the infected mosquitoes confirmed the results obtained by the TaqMan assay and determined that the *OVM+* previously detected in one Obuasi mosquito by the TaqMan specifically corresponds to *P. malariae*. For the Adawukwa *An. coluzzii* population, 6 samples were positive (13.04 %, 6/46) with 3 mosquitoes infected by *P. falciparum* (6.52 %, 4/46), 2 mosquitoes with *P. malariae* (4.35 %, 2/46) and one mosquito presented a mix infection of *P. falciparum* and *P. malaria*e. The nested PCR only confirmed 3 of 6 positive samples infected with *P. falciparum* (6.52 %*)*.

### Insecticide resistance profiles of *An. funestus* (*s.s*.)

**Fig. 1:**
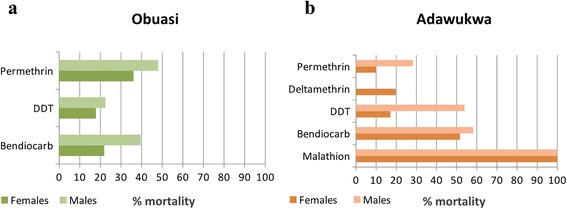
Resistance profile of *An. funestus* (*s.s*.) populations. **a** Obuasi. **b** Adawukwa

A total of 782 adult F_1_*An. funestus* (*s.s*.) mosquitoes from Obuasi and Adawukwa, and 1,851 *An. coluzzii* from Adawukwa were used for WHO insecticide susceptibility assays against four families of insecticides (pyrethroids, carbamates, organochlorines and organophosphates). Females *An. funestus* (*s.s*.) from both locations were highly resistant. In Adawukwa, high pyrethroid resistance levels were observed, with mortality rates of 10.02 ± 3.87 % and 19.76 ± 3.1 % respectively for permethrin and deltamethrin (Fig. 1a). Similarly, high permethrin resistance was also detected in Obuasi, with a mortality rate of 36.11 ± 3.87 % (Fig. 1a). Both *An. funestus* (*s.s*.) populations from Obuasi and Adawukwa were also highly resistant to DDT and bendiocarb, with mortality rates of 17.86 ± 4.51 % and 17.08 ± 5.61 % for Obuasi and Adawukwa, respectively, in the case of DDT; and 21.88 ± 10.7 % and 51.67 ± 4.26 % respectively, in the case of bendiocarb. However, full susceptibility was observed to the malathion with 100 % mortality observed in Adawukwa. The exposure of male *An. funestus* (*s.s*.) to the same insecticides also displayed similar results to those obtained with females.

### Insecticide susceptibility in *An. coluzzii*

**Fig. 2:**
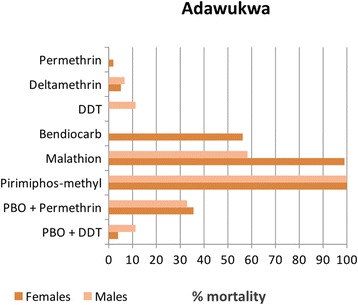
Resistance profile of *An. coluzzii* population in Adawukwa.

To assess whether the resistance observed in *An. funestus* (*s.s*.) was primarily caused by insecticide-based public health control interventions such as LLINs and IRS, we hypothesized that a similar resistance profile could also be observed in other indoor resting malaria vectors notably *An. gambiae* (*s.l*.) To test this hypothesis, *An. gambiae* (*s.l*.) mosquitoes were also collected and insecticide susceptibility bioassay profiles were established. Due to the low number of *An. gambiae* (*s.l*.) collected in Obuasi, only the Adawukwa population was characterized (Fig. 2). Very high pyrethroid and DDT resistance was observed in *An. coluzzii* females, with mortality rates of 1.92 ± 1.92 % and 5.09 ± 0.87 % for the pyrethroids permethrin and deltamethrin, respectively, and with no mortality to the organochlorine DDT. To further assess the extent of permethrin resistance, the LT_50_ was estimated after exposure to permethrin at different time-points. LT_50_ was established as 221.5 min. Resistance to bendiocarb was also detected in adult females with a mortality rate of 56.26 ± 2.24 %. However, similar to *An. funestus* (*s.s*.), the Adawukwa *An. coluzzii* population was also susceptible to the organophosphates, with mortality rates in adult females of 99 ± 1 % to malathion and 100 % pirimiphos-methyl (Actellic).

Synergist PBO suggested the potential role of cytochrome P450 and other mixed function oxidases in the resistance against pyrethroids and DDT in the *An. coluzzii*. For permethrin, a moderate recovery of the susceptibility was observed after PBO pre-exposure, with a mortality of 35.59 ± 7.17 % in contrast with a mortality rate of 1.92 ± 1.92 % obtained without PBO pre-exposure, suggesting that cytochrome P450s may be playing a role in the pyrethroid resistance observed in this populations. However, for DDT, only a minimal recovery of the susceptibility was observed with mortality rate of only 3.92 ± 2.27 % after PBO pre-exposure, suggesting that cytochrome P450 genes have very low or no contribution towards the DDT resistance.

### Transcription profiling of metabolic resistance genes

**Fig. 3:**
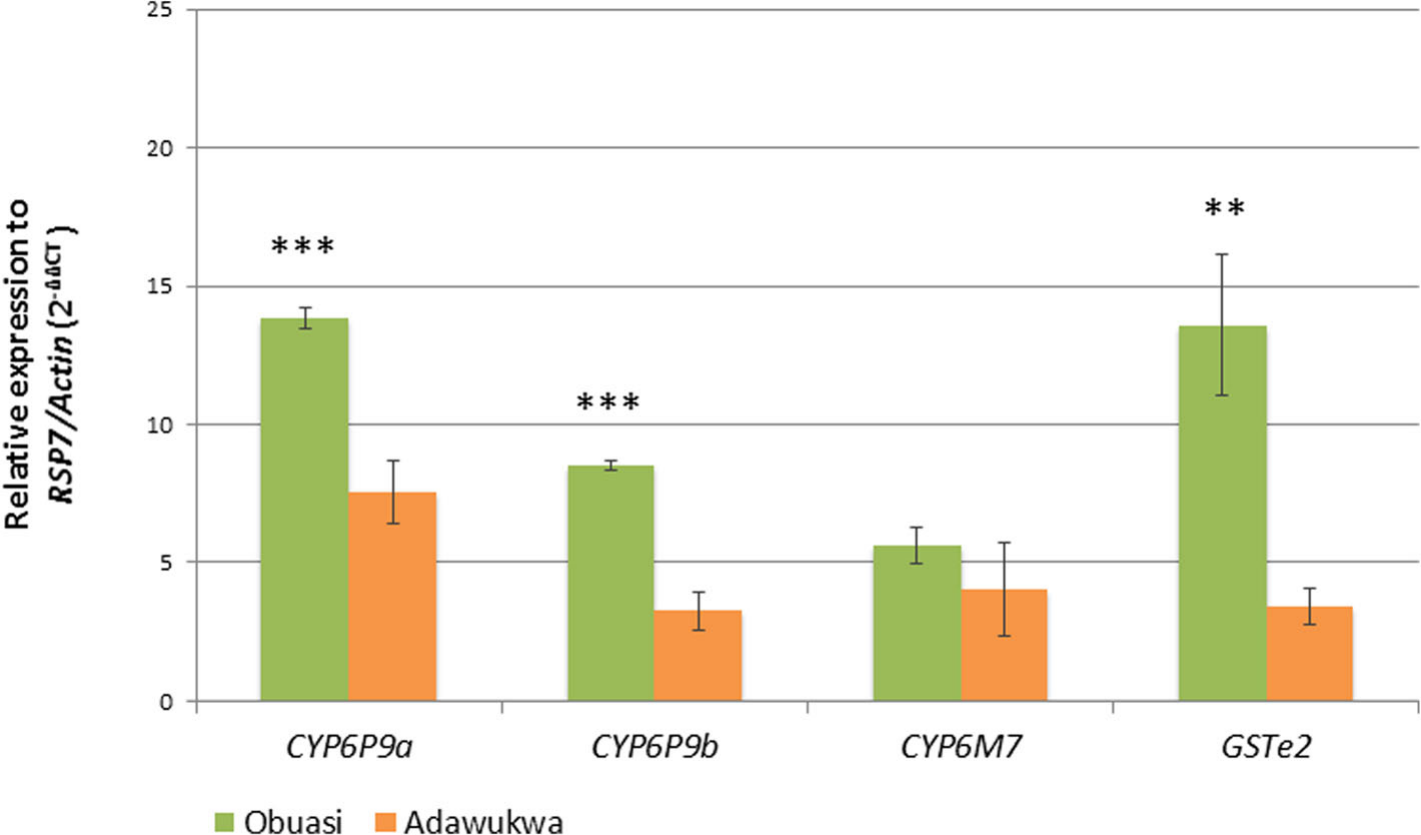
Differential expression of four resistance genes by qRT-PCR between permethrin-resistant *An. funestus* (*s.s*.) mosquitoes from Obuasi (*green*) and Adawukwa (*orange*) and susceptible laboratory strain FANG. Error bars represent standard deviation (*n*  =  3). Significant differences in expression between Obuasi and Adawukwa are indicated: **P < 0.01; ***P < 0. 001

Transcription analysis of the candidate resistance genes *CYP6P9a*, *CYP6P9b*, *CYP6M7* and *GSTe2*, known to confer pyrethroid and/or DDT resistance in *An. funestus* (*s.s*.) [9–11] revealed that these genes are significantly upregulated in the three groups of *An. funestus* (*s.s*.) analyzed from both localities, compared with the susceptible strain FANG. The fold changes (FCs) obtained are summarized in Table 1. Interestingly, a significantly higher upregulation of *CYP6P9a* (*t*_(4)_ = 10.4, *P* = 0.0005), *CYP6P9b* (*t*_(4)_ = 14.6, *P* < 0.0001) and *GSTe2* (*t*_(4)_ = 7.62, *P* = 0.0016) genes was observed in Obuasi mosquitoes compared with Adawukwa. However, no changes were observed on the regulation of *CYP6M7* (*t*_(4)_ = 1.20, *P* = 0.3) (Fig. 3).

**Table 1:** Relative expression by qRT-PCR of four genes from mosquitoes not exposed to insecticides, those that are permethrin-resistant and DDT-resistant, compared with the susceptible strain FANG, in two *An. funestus* (*s.s*.) populations from Obuasi and Adawukwa, Ghana

Locality	Non-exposed mosquitoes	Permethrin-resistant mosquitoes	DDT-resistant mosquitoes
Obuasi			
*CYP6P9a*	8.97 ± 3.71	13.85 ± 0.37	13.91 ± 2.25
*CYP6P9b*	4.13 ± 1.22	8.52 ± 0.18	7.72 ± 1.76
*CYP6M7*	7.84 ± 1.05	5.62 ± 0.65	6.40 ± 0.88
*GSTe2*	16.43 ± 9.93	13.59 ± 2.53	19.47 ± 4.42
Adawukwa			
*CYP6P9a*	5.64 ± 1.91	7.57 ± 1.12	8.73 ± 0.94
*CYP6P9b*	2.57 ± 0.76	3.24 ± 0.69	3.81 ± 0.38
*CYP6M7*	5.31 ± 1.07	4.05 ± 1.69	4.63 ± 1.64
*GSTe2*	6.04 ± 2.46	3.42 ± 0.67	4.20 ± 1.22

### Detection of the L119F-GSTe2 and A296S RDL mutations

**Fig. 4:**
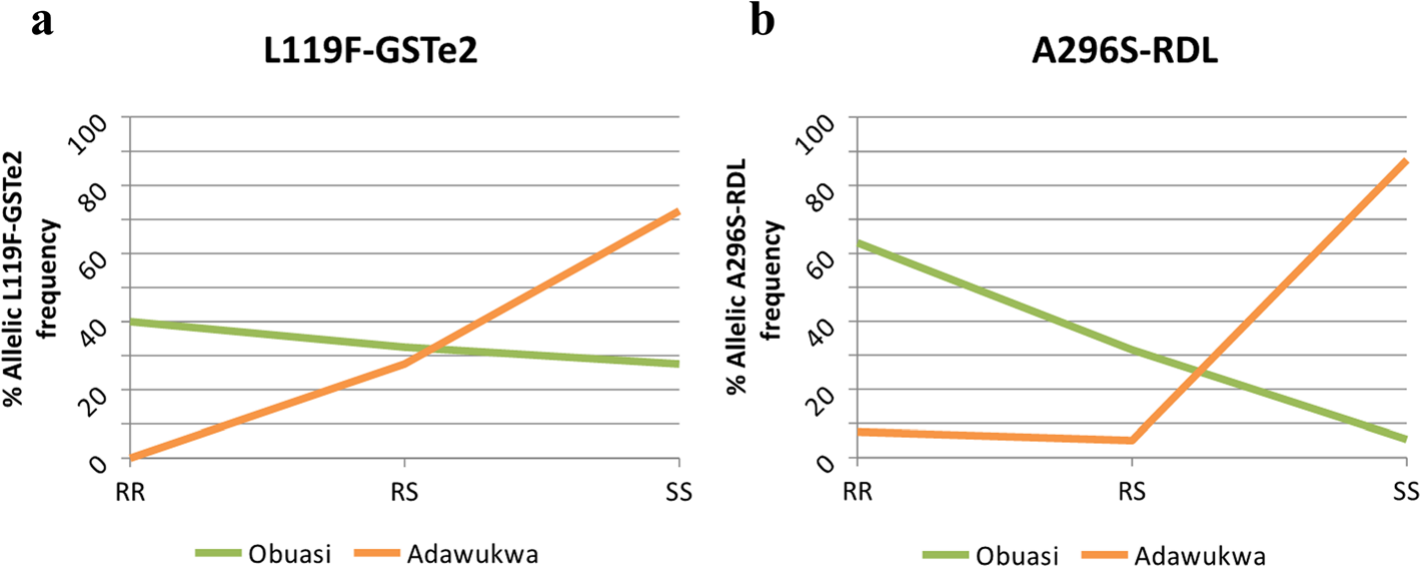
Percentage of frequency of resistant mutations in *An. funestus* (*s.s*.). **a** L119F-GSTe2 related with DDT resistance. **b** A296S-RDL related with dieldrin resistance. *Abbreviations*: RR, homozygote for resistant allele; RS, heterozygote; SS, homozygote for susceptible allele

Genotyping of the L119F-GSTe2 and A296S-RDL mutations with TaqMan using 40 non-exposed F_0_*An. funestus* (*s.s*.) from both locations, showed high frequencies of the 119 F-GSTe2 mutation in the *An. funestus* (*s.s*.) population from Obuasi. Indeed, the 119 F resistant allele is present at a frequency of 56.25 % (45/80) in Obuasi whereas it is four times lower in Adawukwa, with a frequency of 13.75 % (11/80). In Obuasi, 40 % (16/40) of the individuals were homozygote for the 119 F resistant allele (RR) whereas 32.5 % (13/40) were heterozygote (RS), and 27.5 % (11/40) were homozygote for the susceptible allele (SS) L119 (Fig. 4a). In contrast, no homozygote resistant (RR) individual was found in Adawukwa, while only 27.5 % of the individuals (11/40), were heterozygote, RS, and the susceptible genotype L119 (SS) was the predominant (72.5 %, 29/40 individuals) (Fig. 4a). The remarkable contrast in the distribution of the L119F-GSTe2 mutation in these two populations (with a similar high DDT resistance rate) suggests that the DDT resistance in Ghanaian *An. funestus* (*s.s*.) is driven by different molecular mechanisms in addition to the L119F-GSTe2 mutation.

The analysis of the frequency of A296S-RDL mutation in *An. funestus* (*s.s*.) populations from both localities revealed similar pattern to those obtained with L119F-GSTe2 genotyping. High frequency of 296S resistant allele of 78.95 % (60/76) was observed in populations from Obuasi. The 63.15 % of the individuals genotyped were resistant, RR (24/38), 31.58 % were heterozygote resistant RS (12/38) and only 5.26 % were susceptible to dieldrin, SS (2/38) (Fig. 4b). In contrast, a low frequency of the 296S resistant allele of 10 % (8/80) was observed in Adawukwa, where only 7.5 % of the individuals genotyped were RR (3/40), and 5 % were RS (2/40), with the majority of the individuals, 87.5 % (35/40) bearing the susceptible A296 allele (Fig. 4b).

## Discussion

Assessing the insecticide susceptibility in malaria mosquito populations, especially in regions where malaria is endemic like Ghana, is essential for designing suitable resistance management strategies. This study reveals high and multiple resistance to the main insecticides used in public health in two *An. funestus* (*s.s*.) populations from southern Ghana, but probably with significant differences in the underlying molecular basis driving the resistance. The findings of this study provide important information which will help in the implementation of evidence-based insecticide-based control interventions in the present ongoing malaria campaign in the country.

### Contribution of *An. funestus* (*s.s*.) to the malaria transmission in southern Ghana

Beside the confirmation of the significant presence of *An. funestus* (*s.s*.) in southern Ghana as previously reported [2, 7, 12, 24], this study reveals the great role that *An. funestus* (*s.s*.) populations are playing in malaria transmission in Ghana, where it exhibited high *Plasmodium* infection rates above 12 %. The infection rates in *An. funestus* (*s.s*.) in March and November 2014, are considerably higher than previous infection rates recorded in the same area in April 2004 (Obuasi, 1.81 %) [3] and in other locations in Central Ghana as reported in November 2003 (Kintampo, 1.5 %) and November 2005 (Kintampo, 3.7 %) [24]. Furthermore, this infection rate is also high compared with other infection rates recorded across Africa [25]. For example, recent studies performed in *An. funestus* (*s.s*.) populations from Uganda and Malawi, in 2013 and 2014 respectively, reported infection rates no more than 5 % [5, 19], although an *An. funestus* (*s.s*.) population from Tanzania (collected in 2014) exhibited 8 % infection rate [26]. However, Osae et al. [12] has reported a high infection rate of 10 % for *An. funestus* in Adawukwa population collected in 2011–2012 using the ELISA method, supporting the high infectivity level of *An. funestus* (*s.s*.) in these southern Ghana populations. It remains to be established whether the infection levels of *P. falciparum* in *An. funestus* (*s.s*.) have increased in this region recently and if this is due to the multiple insecticide resistance. These high parasite rates, coupled with the fact that *An. funestus* (*s.s*.) was the predominant malaria vector species in locations such as Obuasi, highlights the major role that this species plays in malaria transmission and calls for this vector to be regularly monitored and also targeted by control programmes in the same way as for *An. gambiae* (*s.l*.). The *Plasmodium* infection rate for *An. coluzzii* (6.52 %) confirmed by a nested PCR was half that of *An. funestus* (*s.s*.) (12.5 %), emphasizing the major role of *An. funestus* (*s.s*.) in malaria transmission in southern Ghana.

### Multiple and high resistance in the populations of *An. funestus*

This study has revealed high and multiple insecticide resistance within the *An. funestus* (*s.s*.) populations from southern Ghana, which is a big concern because of the limited number of insecticide classes available for LLINs and IRS. These resistance profiles are notably higher than those reported by Anto and collaborators in northern Ghana [27]. Resistance seems to be increasing in Obuasi when compared to previous observations made in the same area for pyrethroids, DDT and bendiocarb by Coetzee and colleagues in 2005 and 2008 [2, 8]. The increase in resistance levels in *An. funestus* (*s.s*.), coupled with the extremely high pyrethroid and DDT resistance detected in *An. coluzzii* in this area (mortality rates < 5 %), is of great concern for the continued effectiveness of on-going insecticide-based control interventions in Ghana. Similar cases of increased insecticide resistance have also been reported recently in *An. funestus* populations from Malawi [5] and Benin (Djouaka et al., unpublished data) and in *An. gambiae* from Burkina Faso [28, 29]. However, as in other populations of Southern and West Africa, the organophosphate malathion remains fully effective against *An. funestus* (*s.s*.) in southern Ghana, making organophosphates the alternative insecticides for IRS. This recommendation is further compelled by the fact that *An. coluzzii*, sympatric with *An. funestus* (*s.s*.) in southern Ghana, was also fully susceptible to malathion and pirimiphos-methyl, another organophosphate.

### Multiple resistance could be widespread in *An. funestus* from Ghana

In the absence of knockdown resistance mutations in the voltage-gate sodium channel in *An. funestus* [5], this study established that pyrethroid resistance in Ghana populations of this mosquito species is possibly driven by metabolic resistance mechanism. Overall, the role of metabolic resistance is evident by the significant upregulation of the three P450 genes (*CYP6P9a*, *CYP6P9b* and *CYP6M7*) already established as key pyrethroid resistance genes in *An. funestus* (*s.s*.) populations across Africa [9, 10]. The upregulation of the glutathione S-transferase *GSTe2* and presence of DDT resistance mutation L119F-GSTe2 [11] explained the high DDT resistance observed in this population from Ghana. However, the upregulation of the above P450s is considerably lower compared with FCs reported in other populations with similar resistance pattern to pyrethroids, notably in southern Malawi [5]. These different patterns in expression may underlie the fact that other genes or mechanisms may be involved in pyrethroid resistance in southern Ghana. However, the noticeable difference consistently observed in the expression patterns of these genes between the two locations with a higher expression in Obuasi despite a similar resistance level, could be an indication that different underlying resistance mechanisms are driving these resistance profiles. It was recently shown that specific amino acid changes in *CYP6P9a/b* are key in the metabolism activities toward pyrethroids [30]. It is possible that the difference between Obuasi and Adawukwa could be attributed to allelic variation in these P450s in southern Ghana, although other detoxification genes could also be involved.

The significant difference in the underlying molecular mechanism driving resistance between the two locations is further highlighted by the strong contrast in the allele frequencies of the L119F-GSTe2 and A296S-RDL frequencies in populations from the two localities. Despite similar DDT resistance profiles (around 17 % mortality rate for both populations), the *GSTe2* gene was more upregulated in Obuasi than in Adawukwa, but more interestingly, 119 F-GSTe2 resistant allele, which is strongly implicated in DDT detoxification, exhibited higher frequency as well (56.25 *vs* 13.75 %). This suggests that there might be an additional DDT resistance mechanism operating in the Obuasi populations. This difference is similar to what has been recently reported in a southern Africa population in Malawi where despite the presence of DDT resistance there was a complete absence of the 119 F-GSTe2 resistant allele further supporting that genes other than GSTe2 are driving DDT resistance in other populations [5].

The same disparity was observed with A296S-RDL frequency, a mutation involved in dieldrin resistance with higher frequency, almost eight times than obtained in Adawukwa. However, this study did not assess the susceptibility of *An. funestus* (*s.s*.) in southern Ghana to dieldrin. The consistent differences between the population of Obuasi and that of Adawukwa for both metabolic resistance (CYP450s and *GSTe2*) and target-site resistance (RDL mutation) suggest that possible barriers to gene flow exist between these two populations, which will need to be established in future studies. It is also possible that different selective forces have shaped the resistance profiles of these populations. Such differences in the underlying resistance mechanisms should be taken into account when designing suitable insecticide resistance management strategies. Future studies will help to understand the possible barriers to gene flow, the dynamics of the insecticide resistance and the actual contribution of the metabolic resistance genes toward the multiple resistance in these malaria vector populations from Ghana which are separated only by 300 km.

## Conclusions

The high and multiple insecticides resistance in the major malaria vectors reported in this study could endanger the effectiveness of current and future insecticide-based control interventions in southern Ghana. This multiple resistance, probably induced by different molecular mechanisms in different areas southern Ghana stresses the needs to improve the implementation and management of ongoing and future malaria control programs in this region.

## Abbreviations

DDT: Dichlorodiphenyltrichloroethane
DEFA: Department for Environment, Food and Rural Affairs
FC: Fold change
IRS: Indoor residual spraying
LLINs: Long-lasting insecticide-treated nets
LT_50_: Median lethal time
MOP: Malaria operational plan
PBO: Piperonyl butoxide
PMI: President’s Malaria Initiative
WHO: World Health Organization
